# Understanding and Perception of Automated Text Generation among the Public: Two Surveys with Representative Samples in Germany

**DOI:** 10.3390/bs14050353

**Published:** 2024-04-23

**Authors:** Angelica Lermann Henestrosa, Joachim Kimmerle

**Affiliations:** 1 Knowledge Construction Lab, Leibniz-Institut für Wissensmedien, 72076 Tübingen, Germany; j.kimmerle@iwm-tuebingen.de; 2 Department of Psychology, Eberhard Karls University, 72076 Tübingen, Germany

**Keywords:** automated text generation, public attitudes toward AI, ChatGPT impact, automated journalism

## Abstract

Automated text generation (ATG) technology has evolved rapidly in the last several years, enabling the spread of content produced by artificial intelligence (AI). In addition, with the release of ChatGPT, virtually everyone can now create naturally sounding text on any topic. To optimize future use and understand how humans interact with these technologies, it is essential to capture people’s attitudes and beliefs. However, research on ATG perception is lacking. Based on two representative surveys (March 2022: *n*_1_ = 1028; July 2023: *n*_2_ = 1013), we aimed to examine the German population’s concepts of and attitudes toward AI authorship. The results revealed a preference for human authorship across a wide range of topics and a lack of knowledge concerning the function, data sources, and responsibilities of ATG. Using multiple regression analysis with k-fold cross-validation, we identified people’s attitude toward using ATG, performance expectancy, general attitudes toward AI, and lay attitude toward ChatGPT and ATG as significant predictors of the intention to read AI-written texts in the future. Despite the release of ChatGPT, we observed stability across most variables and minor differences between the two survey points regarding concepts about ATG. We discuss the findings against the backdrop of the ever-increasing availability of automated content and the need for an intensive societal debate about its chances and limitations.

## 1. Introduction

Public awareness of the terms automated text generation (ATG), natural language generation (NLG), or large language models (LLM) has grown. The November 2022 release of ChatGPT—a language model developed by the company OpenAI with the capability of generating human-like text in a conversational manner—has fueled the attention to this subfield of artificial intelligence (AI). In detail, NLG is “the subfield of artificial intelligence and computational linguistics that is concerned with the construction of computer systems that can produce understandable texts […] from some underlying non-linguistic representation of information” [1,2]. While the output is always text, the input can vary substantially [3], including flat semantic representations, numerical data, or structured knowledge bases [4]. ATG is a particular type of NLG where natural-sounding text is generated through algorithmic processes with limited human intervention [5]. Already before ChatGPT’s release, these automatically produced texts were no longer distinguishable on a linguistic level from those written by humans [6,7]. What is new, however, is that through the wider availability of ATG technology for a broad population, written text will be more and more automatically created. Moreover, unlike previous customer service chatbots, users interact with open access LLMs such as ChatGPT without a particular purpose. Instead, it is a communicative object in itself [2], providing language translation, summarization, or question answering, all in one tool [8]. Additionally, the underlying technology makes ChatGPT’s output unique compared to automatically written text which existed before. Based on a vast amount of data, LLMs have learned how people use written language and how writing works on a statistical level. This makes applications like ChatGPT specifically different from previous ones, such as those used in automated journalism. Due to the fact that the training data are not stored and the output generation does not follow transparently predetermined rules, it is neither predictable nor fully controllable. Consequently, what defines LLMs and leads to the high quality of their output is also one of their most significant weaknesses and dangers for potential users unaware of the genesis of the resulting text.

Among the first to apply ATG technology systematically were news media organizations that used the more rule-based approaches of ATG to automate news reporting (e.g., Associated Press, Forbes, Washington Post). However, the e-commerce sector was also fast to recognize the potential for automating product descriptions, for instance. This shows that AI-generated content has already existed on the internet for over a decade, but the public perception and awareness of this AI subfield has hardly been investigated. However, the release of ChatGPT threw a spotlight on the technological possibilities of generating human-sounding language. GPT-4 (the underlying model of ChatGPT) and other language models like BERT (Google) or XLNet (Microsoft) now have the potential to revolutionize the way people write, perceive, and use text in all contexts. A prerequisite for realizing the full potential of these AI technologies is that users and readers accept and adopt them [9]. While speech-based applications like voice assistants have been known to many for several years, more creative approaches of NLG on more complex topics and data are not very common and hardly salient in public (e.g., Open Research Knowledge Graph). Currently, there is a discrepancy between small groups of people firmly dealing with the benefits of LLMs in general (ranging from individuals revising their complete working process to companies implementing NLG wherever possible) and a large population for whom this technology still is not a reality. However, unlike other AI subfields, such as in the medical context, ATG is no longer just a niche topic for researchers or developers. No more does it concern only those actively using the technology. The amount of AI-generated content on the internet is growing, with some forecasting that the quantity of synthetically generated content will be up to 90% by 2026 [10]. As AI content will become more prevalent in print media, too, consumers will increasingly encounter automatically written text, often without realizing it, especially online. Therefore, it is crucial and unavoidable that consumers deal with AI authorship and develop pertinent opinions. However, a fundamental issue is the general population’s lack of awareness of automated texts due to inadequate labeling requirements: it is mostly not labeled or only identified by a single byline. To date, this makes it questionable if and to what extent readers have perceived content to be automatically generated. 

In the last decade, many (primarily qualitative) investigations have dealt with journalists’ perspectives on this technology, often through job replacement scenarios [11,12,13,14]. Investigations into readers’ perceptions, attitudes, and beliefs concerning automatically produced content are rare. Consequently, a thoughtful debate about societal, ethical, economic, and juridical implications is late in coming [15]. In short, with the release of ChatGPT, a highly developed technology is accessible to virtually everyone with internet access. At the same time, potential users have not had the time to foster a sharpened awareness of the chances and risks this technology brings.

### 1.1. Automated Journalism

Before the release of ChatGPT, one of the most popular ATG application areas was the automation of news reporting, also known as automated journalism or robot journalism. Several studies have investigated readers’ perceptions and acceptance of automated short news and the novel source cue “AI authorship”. However, the findings concerning the perceived credibility of the content and the author are inconsistent. Some studies found that readers perceived AI-written texts as less credible [16], readable [17], and accurate [18]. Others found AI vs. human written texts to be perceived as equal in expertise, trustworthiness [19], and credibility [20,21,22,23,24]. Moreover, some studies found that AI-written texts were perceived as more credible, objective, and balanced [6,17,25] than human-written texts. However, a meta-analysis across different topics of short news reporting shows that differences, if found, were relatively small [26].

These studies have mainly investigated very number-based topics like weather forecasts, earthquake information, or financial reports, for instance. However, the topics’ content and reporting style might be essential to the findings. The information presented in a weather forecast or a product description leaves little room for interpretation and stylistic creativity. Advantages of algorithms like accuracy or objectivity, as postulated by the machine heuristic [27], might predominate here. Moreover, even if the author is noticed, the person reporting about these number- and data-driven topics might not be of much interest to the reader, which could explain the relatively minor differences found so far. However, even when using a more complex and detailed topic, Lermann Henestrosa et al. [28] found no differences concerning perceived credibility and trustworthiness between an AI and a human author. They discovered at the same time that the AI author was perceived to be less anthropomorphic and intelligent.

### 1.2. Algorithm Aversion

Evidence shows that people have specific expectations toward AI and algorithms in particular contexts. The word-of-machine-effect describes the belief that AI recommenders are more competent than human recommenders in utilitarian vs. hedonistic realms [29]. In addition, the machine heuristic is a cognitive shortcut when ascribing accuracy or lack of bias to an algorithm when performing certain tasks, for instance, a job in online transactions [27,30]. In line with these findings, algorithm aversion describes the consumers’ preference for a human when a task is subjective by nature [31] or concerned with moral decisions because machines are thought to lack a mind and emotions [32]. AI has also been perceived as less competent in giving advice for addressing societal challenges [33]. Applied to AI authorship, Tandoc, Yao, and Wu [23] found a decrease in source and message credibility when the AI was perceived to write non-objectively. In another study, message credibility decreased for both the human and the AI author when the information was presented evaluatively vs. neutrally [28]. With the expanding applicational possibilities of ATGs allowing for the generation of human-sounding text to any possible topic, more research on the perception of AI authorship in different contexts is necessary. 

### 1.3. Surveys on AI and ATG Perception 

An online survey by a local initiative for the media and digital scene in Hamburg, Germany, revealed that in 2018, 49% of the respondents were skeptical toward automated news and robot journalism, and 28% considered it “bad”, while 20% considered it to depend on the topic [34]. Moreover, in a follow-up survey in 2019, 77% of the respondents demanded that automatically produced content be recognizable as such, while only 39% could distinguish between an actual AI-written text and a human-written one [35]. Interestingly, the wording of the questions in this survey suggested that the prevalence of AI-written texts would be realized only in the future.

A survey among American adults in 2022 revealed a general awareness of the public toward AI in daily life, but only three in ten identified all uses of AI provided in the survey correctly [36]. Another representative survey among the German population investigating the general beliefs and attitudes toward algorithms revealed in 2018 that 45% of the respondents could not indicate what an algorithm is. This knowledge gap was accompanied by skepticism toward algorithms, with 79% of the respondents indicating that they preferred human decisions [37]. Also, different applicational fields of algorithms were not known to a majority but became better known in 2022 [15]. In a recent replication, the authors found evidence for a connection between familiarity and acceptance of automized decisions, with decisions being considered more acceptable and the respondents being more familiar with the potential field of application [15]. The term algorithm can stand for a simple mathematical function or a highly complex algorithm for data encryption. However, specific knowledge about certain applications and their underlying technology is often irrelevant to users. But, as ATG is now dominating public debate, it is necessary to investigate what people currently think about this specific AI.

### 1.4. The Current Research

According to the most prominent theory for predicting the acceptance of technology, the technology acceptance model (TAM) [9,38], the adoption of a specific technology is primarily determined by users’ performance expectancy and effort expectancy, which influence the attitude toward using it and finally the intention and actual usage of technology. In addition, the evaluation of automatically produced content might depend on participants’ attitudes toward AI in general. Darda et al. [39] found that a positive attitude toward AI leads to higher ratings for both automated and human-generated content. Furthermore, discussions around AI fields like automated driving or AI in healthcare were not based on tools suddenly accessible to everyone. In these areas, the focus lies more on the decisions made by AI rather than on the underlying technology that leads to them. This is problematic with tools like ChatGPT, where the information provided will probably be judged based on the user’s beliefs about the perceived sources, for instance. However, systematic surveys about people’s beliefs, experience, or knowledge concerning specific AI fields are rare, with the majority dealing with perceptions of AI in general or in the medical context [40,41,42,43] and surveys even leaving out the specific field of ATG entirely [44]. To the best of our knowledge, there is no current investigation specifically on people’s beliefs about ATG or their concepts about its function, responsibilities, or data sources. 

In view of these considerations and research gaps, we posed the following research questions: What attitudes, perceptions, and knowledge does the German population have toward ATG? Have these attitudes, perceptions, and knowledge changed over time, specifically since the release of ChatGPT as a critical event? Additionally, we exploratively investigated people’s behavioral intentions to consume ATG by using several predictors as suggested by the TAM. 

This design made observing a potential change in the data over time possible. Both surveys asked questions about attitude toward ATG, while the second survey included additional questions about ChatGPT to take this event into account as a potential influencing factor. Finally, with its representation of different ages, genders, and educational levels, this study aimed to shed light on differences in the population concerning these subgroups.

## 2. Methods

### 2.1. Sample

The study was conducted in accordance with the guidelines of the Local Ethics Committee of the Leibniz-Institut für Wissensmedien, which approved the study design and methods (Approval number: LEK 2023/022). Written informed consent was obtained from all participants involved in the study. Participants were invited to complete the online survey via the online market research platform Mingle in March 2022 (Study 1) and in June 2023 (Study 2). To assure representativeness in terms of age, gender, and education among the German population over 18 years old, quotas were defined in advance. Responses to all questions were voluntary, but participants were only included in the analyses when they had finished the entire survey. Therefore, exclusion criteria were only premature dropout and missing consent to the use of the data. The survey took 10–15 min in the first and 15–20 min in the second census. Each participation was compensated within Mingle’s internal reward system.

The Pearson’s correlation coefficient with the final sample size of *n*_1_ = 1028 and *n*_2_ = 1013 participants was sufficient to detect correlational effects of *r* = 0.088 with 80% power (alpha = 0.05, two-tailed), according to sensitivity power analysis (G*Power). In other words, correlations greater than *r* = 0.088 could be reliably detected.

The participants in Study 1 were on average *M*_1_ = 46.90 (*SD*_1_ = 15.28) years old (range = 18–73 years). The participants in Study 2 had a mean age of *M*_2_ = 45.58 (*SD*_2_ = 14.27) years (range = 18–69). Table 1 shows the absolute and relative distributions by survey for gender, education, and age.

### 2.2. Measures and Procedure

In the following paragraphs, we describe the measures and procedure of both surveys, as Study 2 was conducted in the same way as Study 1 apart from several additional questions concerning ChatGPT. After giving informed consent and their initial screening with respect to age, gender, and education, respondents were redirected to the survey platform Qualtrics (Provo, UT, USA).

First, self-assessed knowledge about AI in general was captured with a single item from 1 = no knowledge about AI to 5 = comprehensive knowledge about AI. 

Afterwards, participants were briefly introduced to the topic and were presented with a general definition of AI, followed by a section about AI in general.

General attitudes toward AI were assessed by using a 20-item instrument [45]. The measure comprised 12 positively (e.g., “There are many useful applications of AI”) and eight negatively phrased sentences (e.g., “I think AI systems make many mistakes”). These were worded to express a general attitude toward AI systems mainly in society and in the work context. The items were measured on 5-point Likert-scales from 1 = absolutely disagree to 5 = absolutely agree.

The belief in the machine heuristic [27] was used to further measure participants’ assessment of AI. Participants were asked for their degree of agreement, from 1 = absolutely disagree to 5 = absolutely agree, with four adjectives for an AI when performing a task (“unbiased”, “error-free”, “objective”, and “accurate”).

Then, participants were asked if and how often they used different speech-based applications (e.g., voice-based assistants, chatbots, translation systems) in order to examine experience with AI-based writing- and voice-software among the population (5-point scale from 1 = never to 5 = constantly). Moreover, we asked if and how often they used different media types (e.g., TV, radio, magazines) to obtain information about scientific topics. To both questions “ChatGPT” was added as an option in Study 2, which served to analyze further the subgroups with and without ChatGPT experience. Only participants who indicated having used ChatGPT were presented with the attitude toward ChatGPT scale.

Subsequently, we assessed participants’ experience with ATG. Participants were asked if they had ever heard about the fact that AI is able to write texts (Heard about ATG) and if they had ever consciously read an AI-written text (Read an AI generated text), both on 5-point single items from 1 = never to 5 = constantly. If participants indicated with at least item 2 = seldom to have read a text written by AI, they were redirected to an open response field and were asked to state the type of text(s) they had read so far.

At this point in Study 2, a knowledge test about ATG followed. The test covered 15 partly adapted [46] statements, for which participants had to decide whether they were true, false, or if they didn’t know (e.g., “Humans can still easily recognize AI-generated speech as artificial speech”). 

In both surveys, a short description and definition of ATG and automated journalism followed to assure that every participant had at least a basic understanding of the subject matter. To keep it as simple as possible, we consistently used the phrases “AI-written text” or “AI-generated text”.

The definition was followed by a set of self-generated statements to examine people’s conceptions about ATG. The scales referred to the mode of ATG’s function (ATG functionality), the source of the automatically written texts’ content (data sources), the extent of control participants believed a human has over an AI-written text (human control), and who they believed was responsible for the content (content responsibility). Participants rated their perceived likelihood of each item (5-point scales from 1 = not at all to 5 = for certain). 

People’s understanding about ATG functionality was assessed by four statements in Study 1 (e.g., “The AI uses existing words and texts and reassembles them”). In Study 2, the item “The AI calculates the word that is most likely to follow next” was added, as this applies to the LLM underlying ChatGPT. Respondents’ belief about the data sources was assessed by five items in Study 1 (e.g., “The AI produces the content itself, without human intervention”). In Study 2, the item “The AI has been trained with certain content, which it then draws on” was added, as this is a more precise description of ChatGPT’s functionality. To assess people’s belief about human control, we presented three items in Study 1 (e.g., “The human sees the final product and edits it if necessary”). Again, in Study 2, the item “The end product is only indirectly controlled by humans (via built-in rules)” was added. Then, participants were presented with eight entities (e.g., “programmer” or “AI itself”) for which they had to indicate the likelihood that one or the other was responsible for the produced content.

The concepts section was followed by five adapted subscales from the UTAUT-instrument (Unified Theory of Acceptance and Use of Technology) [9] to measure specific attitudes toward AI-written texts. All items were measured on 5-point Likert scales from 1 = absolutely disagree to 5 = absolutely agree. 

Three items were presented concerning performance expectancy (e.g., “I would find AI-written texts useful”), three items concerning effort expectancy (e.g., “I think AI-written texts are clear and understandable”), four items concerning participants’ attitude toward using ATG (AT; e.g., “AI-written texts would make information retrieval more interesting”), three items concerning anxiety (e.g., “AI-written texts are somewhat intimidating to me”), and three items concerning behavioral intentions to consume ATG (e.g., “I intend to read AI-written texts in the future”). To assess participants’ attitude toward what an AI should be permitted to write (permission to write like a human), we added four self-created items (e.g., “AI should be allowed to write about the same topics humans do”).

At the end of the specific attitudes block, participants were asked how likely (5-point scales from 1 = not at all to 5 = for certain) they would be to read an AI-written text on 18 different news media topics (e.g., politics, society, or weather forecasts). Afterwards, the identical list of topics was presented again, asking participants to indicate if they could choose freely by whom they would prefer to read about each topic (“preferably by a human being”, “no preference”, “preferably by an AI”).

In Study 2, participants who had experience with ChatGPT were asked to indicate their agreement to 16 statements addressing their attitude toward ChatGPT (e.g., “I am satisfied with ChatGPT’s answers”; 5-point Likert scale from 1 = absolutely disagree to 5 = absolutely agree).

Finally, independently of their prior experience, all participants in Study 2 were presented with a definition of ChatGPT and were afterwards asked about their lay attitude toward ChatGPT and ATG by indicating their agreement with nine statements (e.g., “I’m optimistic about the impact of automated text generation (e.g., ChatGPT) on society”; 5-point Likert scale from 1 = absolutely disagree to 5 = absolutely agree). 

## 3. Results

### 3.1. General Attitudes toward AI

**Figure 1:**
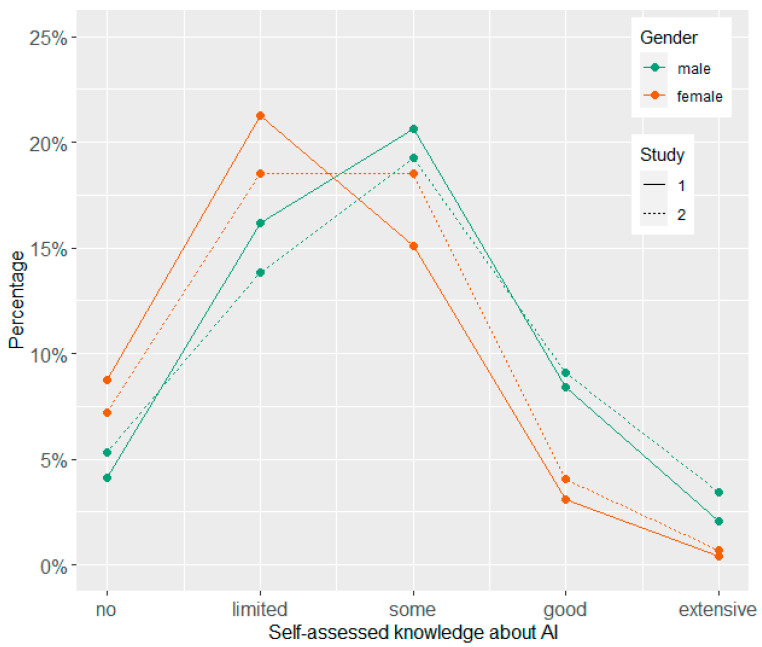
Self-assessed knowledge about AI by gender and study survey.

**Figure 2:**
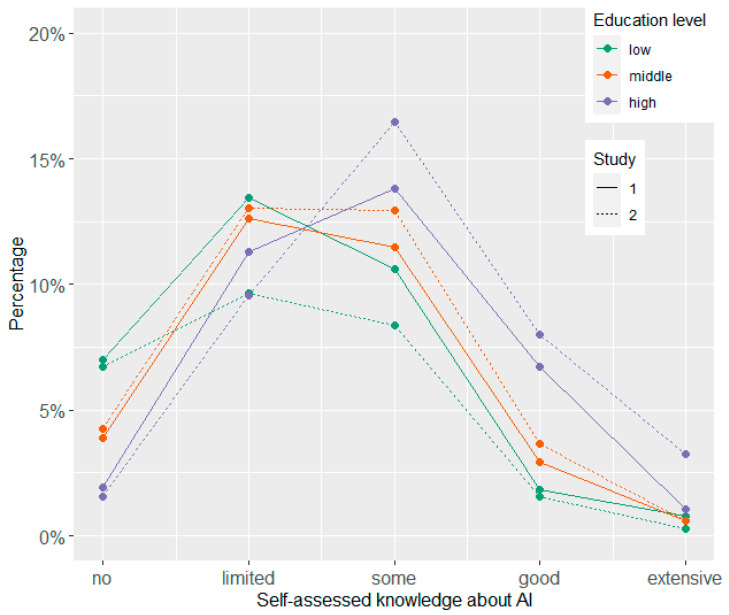
Self-assessed knowledge about AI by education level and study survey.

**Figure 3:**
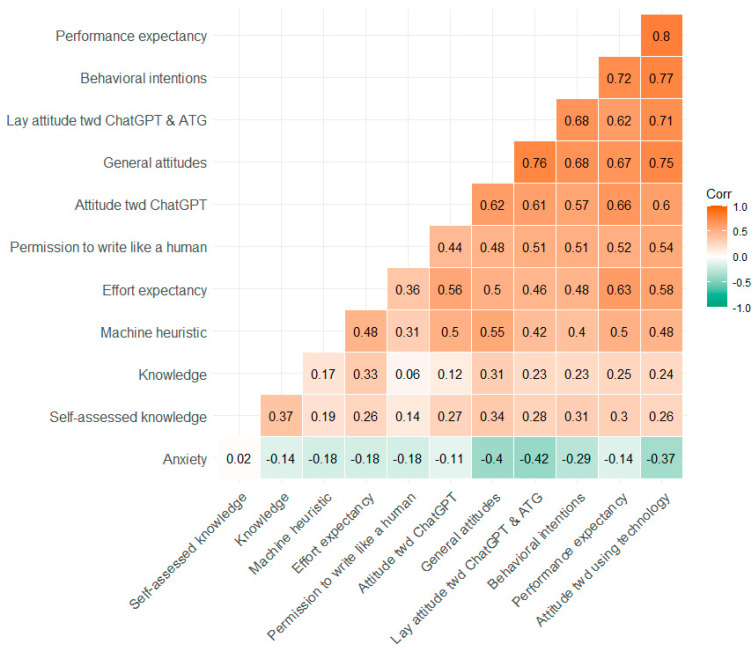
Correlation matrix for all Likert-type variables and the knowledge test in Study 2. The responses of the knowledge test were re-coded for the purpose of the analyses, i.e., the answer option “don’t know” was coded as 0 = false response.

Participants’ answers on the item concerning their self-assessed knowledge about AI are displayed separated by gender and study (Figure 1) and by education and study (Figure 2). Table 2 shows the means, standard deviations, and Cronbach alpha values for all single items and scales by study. In addition, exploratory *t*-tests for independent samples were conducted to compare the two time points. Furthermore, the relationships between the variables in Study 2 are depicted by Figure 3 showing the correlations between all Likert-type variables and the knowledge test (for means and standard deviations of all variables separated by age groups, see Supplementary Table S1).

Due to the poor internal consistency of the scale, which indicates that the items covered different aspects, participants’ answers to each of the four self-created items capturing the permission to write like a human are presented separately. The means and standard deviations by study were as follows: *M*_1_ = 2.98 (*SD*_1_ = 1.06) and *M*_2_ = 3.05 (*SD*_2_ = 1.09) for “AI should be allowed to write about the same topics as humans”, *M*_1_ = 3.70 (*SD*_1_ = 0.96) and *M*_2_ = 3.71 (*SD*_2_ = 1.00) for “AI should present pure facts” (reversely scored in the scale), *M*_1_ = 2.59 (*SD*_1_ = 1.08) and *M*_2_ = 2.67 (*SD*_2_ = 1.12) for “AI may express an opinion in its texts”, and *M*_1_ = 2.82 (*SD*_1_ = 1.07) and *M*_2_ = 2.99 (*SD*_2_ = 1.14) for “The AI may write emotional texts”.

### 3.2. Experience with ATG

**Figure 4:**
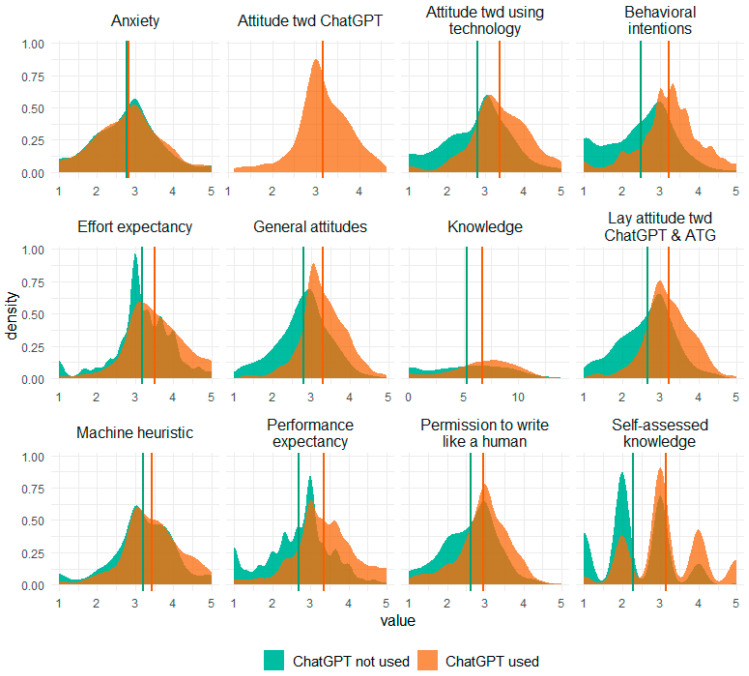
Density distributions of all variables for the subgroups of ChatGPT use in Study 2. Vertical lines represent the means within the subgroups; *n* = 605 for “ChatGPT not used” and *n* = 408 for “ChatGPT used”.

The responses to the questions of whether participants had ever heard of ATG and whether participants had ever read a text written by AI can be seen in Table 3. In addition, the answers specifically regarding ChatGPT use are displayed in this table. A total number of n = 408 participants in Study 2 (40.28% of respondents) indicated having used ChatGPT before and were thus later forwarded to the attitudes toward ChatGPT questionnaire (see below). For distributions of the scales separated for participants who indicated having or not having used ChatGPT, see Figure 4.

### 3.3. Knowledge Test

Concerning the knowledge test in Study 2, 120 (11.85%) respondents did not answer any question correctly while only two (0.002%) reached to answer 14 statements correctly (see Table 2 for mean and standard deviation). For the 15 statements of the knowledge test and the distribution of participants’ responses on each statement, see Table 4.

### 3.4. Concepts

**Figure 5:**
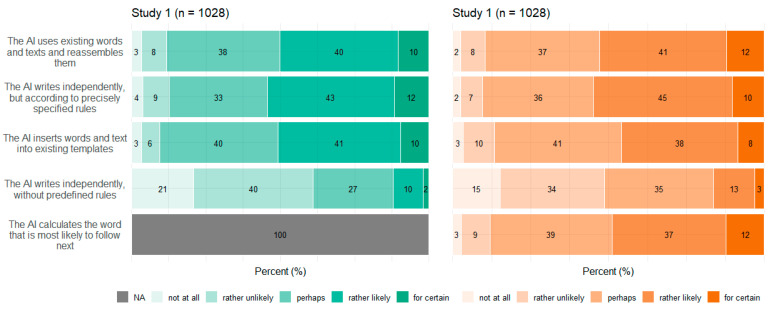
Relative frequencies of participants’ perceived probability for each item regarding ATG functionality by study.

**Figure 6:**
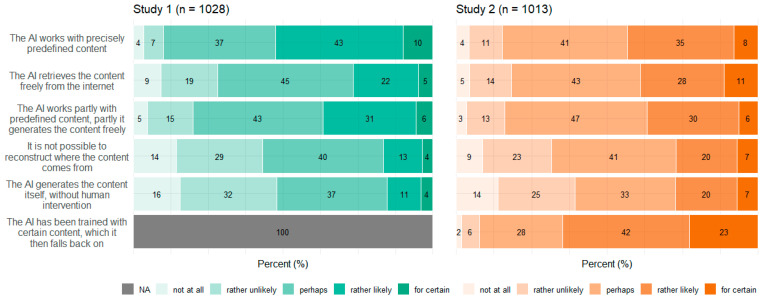
Relative frequencies of participants’ perceived probability for each item regarding data sources by study.

**Figure 7:**
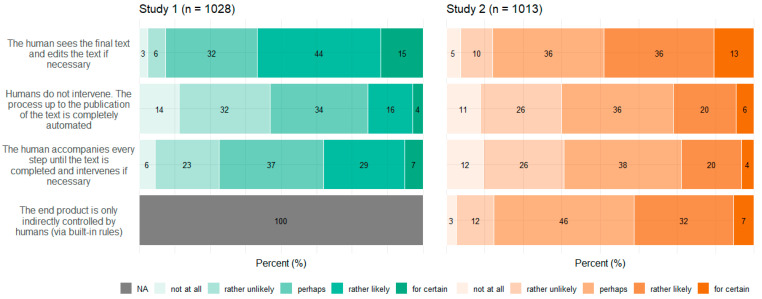
Relative frequencies (percentage) of participants’ perceived probability for each item regarding human control by study.

**Figure 8:**
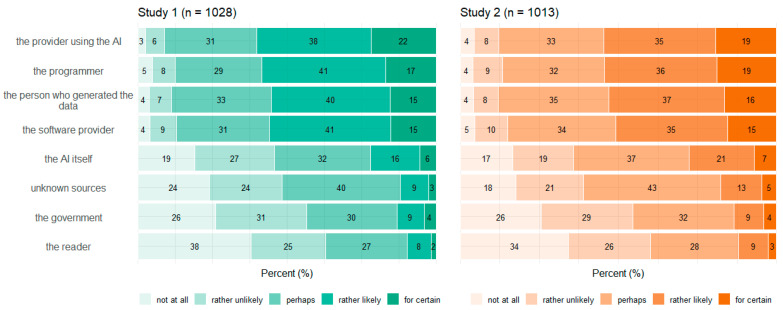
Relative frequencies (percentage) of participants’ perceived probability for each item regarding content responsibility by study.

Figure 5, Figure 6, Figure 7 and Figure 8 show participants’ perceived probabilities toward each item concerning the concepts. Relative answers to each belief about how ATG could potentially work (Figure 5), where the content could come from (Figure 6), how much control the human could have in the process (Figure 7), and who could be responsible for the content (Figure 8) are depicted by study.

### 3.5. Intention to Read AI-Written Texts concerning Journalistic Topics

For participants’ detailed answers on the question “How likely would you be to read AI-written texts on the following topics?” separated by study, see Supplementary Figure S1. Regarding all 18 topics, the proportion of participants answering “perhaps” was between 30–40% for both studies. The option to read an AI written text “for certain” was chosen in equally small proportions in both studies, with the highest values for product descriptions and weather forecasts. The proportion of participants indicating to be “not at all” willing to read an AI-generated text to the presented topics reached between 8–30% in Study 1 and 12–26% in Study 2, with the highest proportion of rejection for the topic “opinion” in both time points. 

Concerning the question “If you had a choice, who would you rather be informed by about the following topics?”, detailed response distribution across the 18 topics is depicted in Supplementary Figure S2. At both time points and regarding all topics, the percentage of people who chose “preferably by an AI” did not reach a majority. The proportion of participants preferring an AI author was between 3–21% in Study 1 and 5–20% in Study 2, depending on the topic. On some topics, respondents expressed a clear tendency to prefer a human author (e.g., opinion, health, politics), whereas on some topics the option “no preference” was selected more frequently (e.g., sports reports, advertisements, stock reports).

### 3.6. Specific Attitudes toward ATG

In Study 2, participants who indicated having used ChatGPT were asked about their attitudes and their experience with this tool. The answer distributions regarding each item are depicted in Table 5. Furthermore, all participants indicated their attitudes toward ATG and ChatGPT on a more general level (Table 6).

### 3.7. Exploratory Analyses

**Figure 9:**
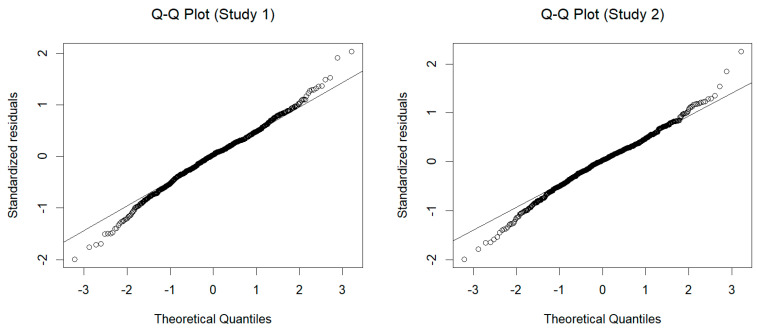
Q-Q plots of residuals to assess normality in the regression models predicting behavioral intention to consume ATG for both studies.

In both studies, we measured participants’ intentions to read AI-written texts and to use ATG technology. Due to the release of ChatGPT in November 2022, we were able to ask more specifically for attitudes toward ChatGPT and ATG in Study 2. Therefore, we conducted two explorative multiple regression analyses to predict people’s behavioral intentions to consume ATG. Figure 9 depicts Q-Q plots for evaluating residual normality in the models for both studies. The plot shows that the residuals follow the theoretical quantiles of the normal distribution well around the mean, with some deviation at the tails of the distribution. This deviation is expected (and commonly seen) since the theoretical normal distribution ranges from minus infinity to infinity, which is, of course, not true for our measure. 

We adopted the common supervised machine learning principle k-fold cross-validation, which helps to estimate the predictive accuracy of a regression model. Using cross-validation, the data set is divided into a training (used for model training) and a test set (also hold-out set, used to evaluate the model performance). The purpose of this approach is not to fit the model to the entire sample but only to a part of it (training set) and thus to test whether the model can be generalized to the unseen hold-out set. Furthermore, the training set is divided into k equal-sized folds on which the statistical model is iteratively developed and fitted, leaving each fold out in turn. This process is repeated k times, with each fold being used as the validation set once. Therefore, cross-validation is a robust and reliable method to reduce the risk of model overfitting and serves the generalizability of the regression results to unseen data [47,48,49]. 

According to the TAM [9], the intention to use a technology is determined by people’s attitude toward using it, which is in turn influenced by the performance expectancy and effort expectancy. Therefore, we added these variables to the models. We also aimed to investigate the predictive contribution of several other variables, such as the general attitude toward AI or participants’ prior experience with ChatGPT, as relationships between these variables were found before [39,50]. 

**Figure 10:**
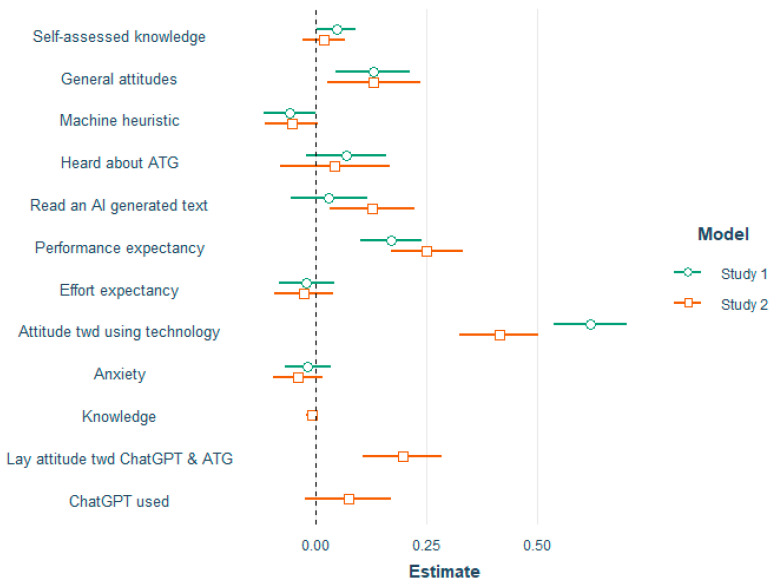
Coefficients of the predictors of behavioral intention to consume ATG for both models (Study 1 & Study 2). Whiskers represent the 95% CI.

Each survey data set was split into a training (75%) and a hold-out set (25%). A regression model using *k* = 5-fold cross-validation was used in each training set. This process involved training the model in four subsets and evaluating it in the remaining subset in a rotating fashion. Finally, the performance on the fitted model was assessed in the hold-out sample. The predictors of the respective models as well as their corresponding coefficients can be seen in Table 4 and are illustrated in Figure 10. Results indicated that both models explained a substantial proportion of variance, with R^2^_1_ = 0.66 and R^2^_2_ = 0.66. Together with the small average magnitudes of errors, RMSE_1_ = 0.48 and RMSE_2_ = 0.53, and mean absolute errors, MAE_1_ = 0.37 and MAE_2_ = 0.43, the fitted models were highly accurate in their predictions. In both models, as expected, performance expectancy and attitude toward using ATG significantly contributed to predicting behavioral intentions, but effort expectancy did not. Moreover, in Study 2, the added predictor lay attitude toward ChatGPT and ATG was a significant predictor (see Table 7). 

## 4. Discussion

What society expects from technological development, which information people may need to understand and adopt innovations, or whether the attitudes tend to be positive or negative should be investigated parallel to the developmental process. Awareness of people’s perceptions and ideas regarding technologies that are ever more present is essential for a meaningful debate about developments and adequate information for laypeople. However, discussions such as those that have been held publicly about deep fakes or facial recognition technology are missing regarding ATG, as public perception is currently largely shaped by one new tool. Even if many people are currently not aware of where ATG is already being used or cannot distinguish between human and AI-written texts, research into attitudes and possible concerns is essential, as these influence trust in and the handling of texts authored by AI. Moreover, knowledge gaps need to be revealed to target misconceptions. We addressed this research gap by investigating the German population’s current beliefs, concepts, and attitudes toward ATG. Data from two representative surveys conducted in 2022, before the release of ChatGPT, and 2023, after the sudden media focus on NLG developments, were collected to gain insights into the current state and potential changes over time.

### 4.1. Public Awareness of ATG 

Without a doubt, the hype surrounding ChatGPT has drawn attention to this field of AI. However, a survey among the U.S. population finding that 42% of Americans had in March 2023 heard nothing at all about ChatGPT revealed that this did not reach all sections of the population equally quickly [36]. While a third of respondents in Study 1 presented here indicated never having heard that AI can write texts, this proportion decreased substantially to 16% in Study 2. In contrast, the proportion of people indicating never having read an AI-written text barely dropped from 56% to 49% between the two polls. Apparently, the coverage of ChatGPT has led people to become more concerned with this technology. Though we cannot retrospectively measure participants’ actual experience with ATG, the evidence still reflects a remarkable lack of awareness of the presence of ATG. Automatically produced content has been present in automated journalism for over a decade (unknown to a majority, as the knowledge test indicated), and automatic product descriptions in online shops are also not new phenomena. Similarly, a German survey by DIW Berlin revealed that many people are unaware of AI in their work contexts: when indirectly asked about AI at work, nearly twice as many respondents indicated working with AI compared to when they were directly asked [51]. It is important to consider that the criteria for what constituted AI are also shifting. The futuristic, distant, and complex image many associated with AI certainly differs from their image of a simpler translation algorithm. Furthermore, an in-depth understanding of technology is not decisive for people to apply it. Nevertheless, future research should constantly adapt to the given circumstances and accompany technological developments.

### 4.2. Self-Assessed Knowledge about AI

Concerning self-assessed knowledge about AI, we observed only small shifts from Study 1 to Study 2. However, there were differences on gender and education levels. Men rated their knowledge higher slightly more often than women, while women indicated more frequently than men having limited or no knowledge. In addition, the higher the educational level, the higher the proportion was of participants who indicated some or good knowledge about AI in general. Similar to a survey from Bertelsmann Stiftung [37], we found that people who indicated knowing more about AI in general had a more positive attitude toward it. Moreover, we also found a positive but rather moderate correlation with the performance in the knowledge test. An educational advantage could enable people to more effectively determine how to use technology to their advantage.

### 4.3. Attitudes toward ATG

We explored significant differences between the two time points on most scales that were surveyed twice. The general attitudes toward AI and the belief in the machine heuristic decreased in Study 2, reflecting a less positive attitude and a slightly diminished belief in the accuracy and objectivity of AI. However, the effect sizes do not allow any conclusion of substantial practical relevance. Rather, this study observed stability across the concepts over time.

Furthermore, high positive correlations occurred among the attitude scales (see Figure 3): The more positive the attitude toward AI, the more favorable was the attitude toward ChatGPT and ATG as well, a relationship also found among a student sample in Arabic countries [52]. However, more intriguing is the positive relationship between the machine heuristic (i.e., the belief that an AI is objective, accurate, neutral, and unbiased) and the attitude scales, as they suggest that predispositions can predict reactions to specific technologies. Furthermore, anxiety was negatively correlated, especially with lay attitude toward ChatGPT and ATG and the general attitudes toward AI, a pattern already found before [53] and in other cultural contexts [52]. More research is needed to address concerns and worries to specifically enable people to deal with ATG appropriately. Tool-specific strengths and weaknesses can get lost in public debates about “generative AI”. Educational gaps can either be bridged or widened by ATG technology, particularly when they are primarily used by groups that already benefit from new developments more than others. This underscores the necessity of widespread and transparent information about the potentials and limits of ATG. 

### 4.4. Knowledge Regarding ATG

The knowledge test conducted in Study 2 revealed some detailed insights into potential misconceptions and knowledge gaps. Almost half of the participants incorrectly believed that the quality of AI-created texts depends only on the training data set. That many also believed that language models have learned to understand language like a human. Of course, these two items require a high level of technological understanding. However, the distribution of answers could reflect a misconception about the fundamental function of AI and ATG: It is a popular misunderstanding that artificial and human intelligence work in the same way. Whereas AI developers aim to imitate human intelligence orienting on the mere results, the technological process of reaching these (seemingly) intelligent results can differ significantly from human cognitive or physical processes. The statements correctly answered by a considerable proportion of participants covered more general aspects with no need for intense technical understanding (e.g., “The statements of language-generating AIs are always correct”). Overall, a high proportion of participants responded “don’t know” to each statement, reflecting the heterogeneous level of knowledge in the population but also emphasizing the need for more information and explanation about ATG. 

### 4.5. ATG-Related Concepts

Regarding the four concepts relating to ATG, respondents had to assess the likelihood that each statement applied, as the concepts covered different possibilities rather than hard facts. However, within each item of the concepts function of ATG and data source, a high proportion of participants chose the option “perhaps”. No clear tendency toward single statements was observed within any of the four concepts. Still, a substantial portion of participants perceived most ideas as “rather likely” or “rather unlikely”. Only a small fraction committed to the answer options “for certain” or “not at all”. In Study 2, an item was added to three of the concepts, to cover ChatGPT’s mechanisms. Concerning function of ATG, the added item “The AI calculates the word that is most likely to follow next” (which describes the function very simply) was perceived to be nearly equally likely as the other options. Only the statement that the AI writes independently was perceived as the least probable option. Concerning data source, participants perceived it to be most likely that the AI would have been trained with certain content, which it then falls back on. Moreover, the items “The AI retrieves the content freely from the internet” as well as “It is not possible to reconstruct where the content comes from” and “The AI generates the content itself” were perceived to be likely more often than in Study 1. 

Overall, the high frequency of the answer option “perhaps” along with the result that no statements sticked out with a clear tendency of being favored speak for a great uncertainty concerning the underlying mechanisms of ATG. Similarly, a current survey on ChatGPT found high proportions of indecision, too [54]. Some items also reflected different existing technological approaches of ATG, which do not necessarily contradict each other. It is also unrealistic that users would be comprehensively informed about all the specific underlying technologies. Nevertheless, a basic understanding of the potentials and limitations of ATG is crucial for a realistic assessment of its application and use. Furthermore, with the release of tools available for people with every conceivable level of knowledge, benchmark studies such as the present one can provide information about misconceptions and possible weaknesses of technologies that must be addressed. 

### 4.6. Preferences for Human Authorship

The current study shows that the broad field of topics that falls under “news” has to be examined in a more sophisticated way, since people seem to have topic-specific preferences. Similar to what “algorithm aversion” [31] predicts, respondents are more likely to read AI-written content about objective and impersonal topics (e.g., traffic news, weather forecasts, or product descriptions). In contrast, participants particularly refuse to read AI texts about genuinely human-centered topics [55] (e.g., society, culture, or politics). When directly asked for author preferences, participants prefer human authorship across all 18 topics, even with slight shifts toward human preference in Study 2. In both surveys, the notable number of participants selecting “perhaps” or “no preference”, along with many indicating they have never read AI-written texts, suggests uncertainty or a lack of imagination about what AI-created content entails. The results are remarkable against the background of a tool that aims to completely imitate written human language in all areas. At the same time, in the context of science communication, two studies suggest that readers perceive a human and an AI author as equally credible [28] or only slightly less credible [56]. However, the current technical possibilities seem to differ from what people want and expect from ATG. Future studies on the actual usage of ChatGPT will hopefully shed light on what people use it for indeed.

### 4.7. Limitations

Since this survey approach concentrated on depicting people’s attitudes at two separate points in time, the two distinct samples do not allow for concluding inter-individual changes, thus rendering them as merely two snapshots. Furthermore, the second survey took place relatively shortly after the publication of ChatGPT. The German population might not have had enough time to get in touch with this tool, and different population groups did not have access to it equally quickly. As we are one of the first to systematically investigate people’s attitudes and concepts regarding this specific subfield of AI, our survey captures rather general aspects. This also means that the aspects asked for, such as previous use of ChatGPT, were only recorded very superficially, meaning that large variance can be assumed at the individual level. Of course, the variety of technological implementations and possible applications could not be covered here by any means. Therefore, the cautious approach only allows for preliminary conclusions and should be specified in future studies. 

### 4.8. Implications and Future Directions

The present study revealed that much of the German population has not yet had conscious contact with ATG technology, though the release of ChatGPT seemed to have an impact, at least on people’s general awareness. On average, extreme attitudes were not observed. Whether this expresses a balanced attitude toward this specific AI application remains doubtful. The large proportion of answer selections expressing uncertainty in numerous scales and concepts instead shows that we confronted the samples with a relatively unknown field. It suggests a situation in which this type of AI flows into the most diverse areas at breakneck speed while facing a public widely naïve to it. Nevertheless, a certain amount of basic knowledge about ATG is present in some respondents, but the need for education also became apparent. Since our second survey took place only seven months after the release of ChatGPT and our results indicate that a significant part of the population still did not have the chance to get used to this technology, it remains to be observed how people’s attitudes will change in the long run. Other analyses have made it clear that people with experience with this technology tend to have more positive attitudes and are more open to the use and consumption of ATG. Therefore, skepticism and unease should be encountered with broad knowledge and competence building. 

The possible applications outside of journalism are as diverse as they are unexploited, ranging from creative writing tasks to highly formalized and standardized content generation and from informal interpersonal communication to academic writing. As ATG can be used in virtually any setting where text is required, future studies will have to cover a broad range of topics but also delve deep into specific subject matters. The diversity of technical approaches will lead to an improvement not only in ATG but also in general AI (cf. [57]). Given that the underlying data will be irrelevant for readers and that providers rarely label AI-generated text, public discussion should approach ATG with a different focus. Similarly, for AI and algorithms in general, knowledge and competence building that reaches the general population [15] are necessary for ATG. Only with a sharpened understanding of chances and risks can users learn to handle ATG in an informed way and participate in a debate about potential regulations and the shaping of the technologies. The present study contributes to a basic understanding of current concepts and attitudes, which could serve as a benchmark for further studies. The results can also serve as a first indication for various types of stakeholders, who, for example, should orient on the desire for clear labeling and address misconceptions when letting people interact with AI-generated texts. Also, future research needs to understand what people expect from ATG and which misconceptions should be addressed. Speaking for the German population, with the current state of knowledge and awareness, the ground is paved for misattribution, disinformation, and credibility issues in journalism, while at the same time, building competence in informal and formal learning contexts cannot be fully exploited. 

## Figures and Tables

**Table 1:** Absolute and relative (in percent) numbers of participants in Studies 1 and 2 by gender, education level, and age group. The German education system differentiates between qualifications after the 9th (low), 10th (middle), and 12th or 13th grade (high). Respondents indicating having no degree (*n*_1_ = 13, *n*_2_ = 4) are included in the low level.

	Study 1		Study 2	
	*n*	%	*n*	%
Gender				
male	527	51.26	516	50.94
female	499	48.54	495	48.86
diverse	2	0.19	2	0.20
Educational level				
low	346	33.66	270	26.65
middle	324	31.52	349	34.45
high	358	34.82	394	38.89
Age group				
18–29	184	17.90	188	18.56
30–39	182	17.70	188	18.56
40–49	175	17.02	188	18.56
50–59	232	22.57	248	24.48
>60	255	24.81	201	19.84

**Table 2:** Mean, standard deviation, and Cronbach alpha for all variables (number of items in brackets) by study and differences in means (Δ*M*) with Cohen’s d, tested using exploratory unpaired *t*-tests; * *p* < 0.05, ** *p* < 0.01, *** *p* < 0.001. All variables except for knowledge were measured on 5-point scales.

	Study 1 (*n* = 1028)		Study 2 (*n* = 1013)				
Variable	*M*	*SD*	*M*	*SD*	α	Δ*M*	*d*
Self-assessed knowledge (1)	2.53	0.94	2.64	1.00	-	0.11 *	0.11
General attitudes (20)	3.16	0.64	3.00	0.69	0.92	−0.16 ***	0.24
Machine heuristic (4)	3.46	0.73	3.30	0.80	0.79	−0.16 ***	0.21
Knowledge (15)	-	-	5.88	3.37	-	-	-
Performance expectancy (3)	2.85	0.83	2.96	0.90	0.81	0.11 **	0.12
Effort expectancy (3)	3.21	0.74	3.31	0.79	0.71	0.09 **	0.12
Attitude twd using ATG (4)	3.01	0.80	3.03	0.87	0.79	0.02	-
Anxiety (3)	2.69	0.83	2.80	0.86	0.71	0.11 **	0.13
Behavioral intentions to consume ATG (3)	2.77	0.83	2.78	0.92	0.67	0.01	-
Permission (4)	2.67	0.72	2.75	0.73	0.62	0.08 *	0.11
Attitude twd ChatGPT (16)	-	-	3.16	0.61	0.89	-	-
Lay attitude twd ChatGPT & ATG (9)	-	-	2.88	0.73	0.82	-	-

**Table 3:** Relative frequencies (in percent) of answers on the items regarding ATG experience and ChatGPT use by study. * Answer options seldom, occasionally, often, and constantly were merged.

		Percentage (%)	
Item	Answer Option	Study 1 (*n* = 1028)	Study 2 (*n* = 1013)
Heard about ATG	Never	32.49	16.09
	Seldom	17.41	10.46
	Occasionally	32.68	32.77
	Often	15.37	27.44
	Constantly	2.04	13.23
Read an AI-generated text	Never	56.52	48.86
	Seldom	21.01	17.28
	Occasionally	16.63	22.01
	Often	5.06	8.19
	Constantly	0.78	3.65
General ChatGPT use	Never	-	64.76
	At least seldom *	-	35.24
ChatGPT use for scientific information	Never	-	65.25
	At least seldom *	-	34.75

**Table 4:** Relative frequencies (in percent) of the correctness of the answers on the knowledge test and correctness of each statement (True/False) in Study 2 (*n* = 1013).

		Percentage (%) of Answers		
Statement	Correctness of Statement	Correct	Incorrect	Unknowing
Pupils can have their homework created with the help of speech-generating AI	True	59.13	11.55	29.32
2. There are different types of models used for ATG	True	57.16	6.81	36.03
3. ChatGPT has been trained with millions of texts from the web, social media, online forums, newspaper articles and books	True	56.37	6.32	37.31
4. The statements of language-generating AI are always correct	False	56.27	11.45	32.28
5. Access to language-generating AI is reserved for certain groups of people (e.g., scientists)	False	52.52	12.14	35.34
6. Speech-generating AI responses may be biased (e.g., racially) based on the data they were trained on	True	46.40	12.83	40.77
7. A chatbot can answer the question ‘Will it rain tomorrow?’ correctly with a high probability	True	45.01	21.42	33.56
8. AI language models (e.g., ChatGPT) calculate for their answers which word is most likely to come next	True	44.52	10.56	44.92
9. Humans can still easily recognize AI-generated speech as artificial speech	False	43.53	23.30	33.17
10. AI language models can intentionally lie and spread false information	False	28.43	31.00	40.57
11. Humans can answer questions about a read text better than AI systems	False	26.16	33.17	40.67
12. AI language models (e.g., chatbots) can give good answers because they have learned to understand language like a human	False	20.53	47.29	32.18
13. The automatic generation of texts has been used in journalism for over 10 years	True	20.24	20.42	59.33
14. Texts created by AI must be legally marked as such	False	17.67	39.59	42.74
15. The quality of the texts created by AI depends only on the training data set used	False	14.41	49.06	36.53

**Table 5:** Relative frequencies (in percent) of participants’ agreement to each item regarding attitude toward ChatGPT in Study 2 (*n* = 408).

	Percentage (%) of Answers				
Item	Absolutely Disagree	Rather Disagree	Undecided	Rather Agree	Absolutely Agree
When I’m unsure with something I would rather trust ChatGPT than me.	17.40	25.49	34.31	18.38	4.41
2. The answers ChatGPT provides are as good as the answers a highly competent person would give.	8.82	19.12	42.16	22.79	7.11
3. I will recommend ChatGPT.	5.88	9.80	41.42	29.41	13.48
4. I will continue to use ChatGPT.	3.68	4.90	40.20	33.82	17.40
5. I trust ChatGPT.	7.11	14.71	45.83	24.51	7.84
6. I still prefer receiving answers and texts from a human.	2.45	11.03	42.89	27.45	16.18
7. I like ChatGPT.	4.90	6.37	46.81	28.92	12.99
8. I know how to use ChatGPT to get the results I need.	5.88	13.97	39.95	30.88	9.31
9. I doubt the answers ChatGPT gives me.	5.15	21.57	47.79	18.87	6.62
10. I can rely on ChatGPT’s answers when it comes to decisions.	8.33	15.44	44.61	24.26	7.35
11. I believe in ChatGPT’s answers even if I can’t be sure they’re right.	8.82	21.32	43.63	21.57	4.66
12. I’m satisfied with ChatGPT’s answers.	4.41	8.33	46.08	32.11	9.07
13. ChatGPT uses appropriate methods to generate its answers.	3.19	6.62	41.91	37.99	10.29
14. ChatGPT provides me all information I need.	6.37	11.76	45.83	27.70	8.33
15. ChatGPT’s answers meet my expectations.	4.17	11.03	42.16	33.82	8.82
16. ChatGPT’s answers are unusable.	18.63	29.41	34.07	14.71	3.19

**Table 6:** Relative frequencies (in percent) of participants’ agreement to each item regarding lay attitude toward ATG and ChatGPT in Study 2 (*n* = 1013).

	Percentage (%) of Answers				
Item	Absolutely Disagree	Rather Disagree	Undecided	Rather Agree	Absolutely Agree
There should be a labelling requirement for AI-generated texts.	3.26	4.24	20.04	28.13	44.32
2. Policymakers should make precise rules on where automated text generation may be applied.	5.53	8.09	28.33	31.00	27.05
3. I feel uneasy with ChatGPT.	13.03	18.26	34.55	19.64	14.51
4. I intend to try ChatGPT.	19.25	14.31	30.21	22.31	13.92
5. I find ChatGPT dangerous.	11.35	19.55	38.50	18.85	11.75
6. I understand how ChatGPT works.	6.42	12.34	40.28	30.90	10.07
7. ChatGPT should be used for scientific information, too.	11.85	14.02	40.08	24.68	9.38
8. ChatGPT should be prohibited.	26.65	26.36	28.83	9.97	8.19
9. I’m optimistic about the impact of automated text generation (e.g., ChatGPT) on society.	13.43	17.57	42.65	20.63	5.73

**Table 7:** Coefficients of the multiple regression analyses with cross-validation for predicting behavioral intentions to consume ATG in Studies 1 and 2. Heard about ATG, read an AI-generated text, and ChatGPT use were included in the models as dummy variables with 0 (not heard, not read, and no usage) serving as the reference group.

	Study 1			Study 2		
	*b*	*SE*	*p*	*b*	*SE*	*p*
Self-assessed knowledge	0.05	0.02	0.031	0.02	0.02	0.409
General attitudes	0.13	0.04	0.003	0.13	0.05	0.014
Machine heuristic	−0.06	0.03	0.050	−0.05	0.03	0.084
Heard about ATG	0.07	0.05	0.132	0.04	0.06	0.480
Read an AI-generated text	0.03	0.04	0.480	0.13	0.05	0.008
Performance expectancy	0.17	0.04	<0.001	0.25	0.04	<0.001
Effort expectancy	−0.02	0.03	0.544	−0.03	0.03	0.438
Anxiety	−0.02	0.03	0.517	−0.04	0.03	0.177
Attitude twd using ATG	0.62	0.04	<0.001	0.41	0.05	<0.001
Knowledge	-	-	-	−0.01	0.01	0.315
Lay attitude twd ChatGPT and ATG	-	-	-	0.20	0.05	<0.001
ChatGPT use	-	-	-	0.07	0.05	0.130

## Data Availability

The data used in the study can be made available on requests addressed to the corresponding author.

## Supplementary Materials

The following supporting information can be downloaded at: https://www.mdpi.com/article/10.3390/bs14050353/s1, Figure S1: Relative frequencies (percentage) of participants’ perceived probability on how likely they would be to read 18 topics written by AI, by study; Figure S2: Relative frequencies (percentage) of participants’ author preference regarding 18 topics, by study; Table S1: Means and standard deviations of all variables separated by age group and study.
