# Supplementary material for: Understanding and Perception of Automated Text Generation among the Public: Two Surveys with Representative Samples in Germany
Source: Behav Sci (Basel). 2024 Apr 23;14(5):353. doi: 10.3390/bs14050353 (PMC11118015; doi:10.3390/bs14050353)
Supplement: Supplementary file 1 [file behavsci-14-00353-s001.zip › behavsci-2905207-supplementary.pdf]

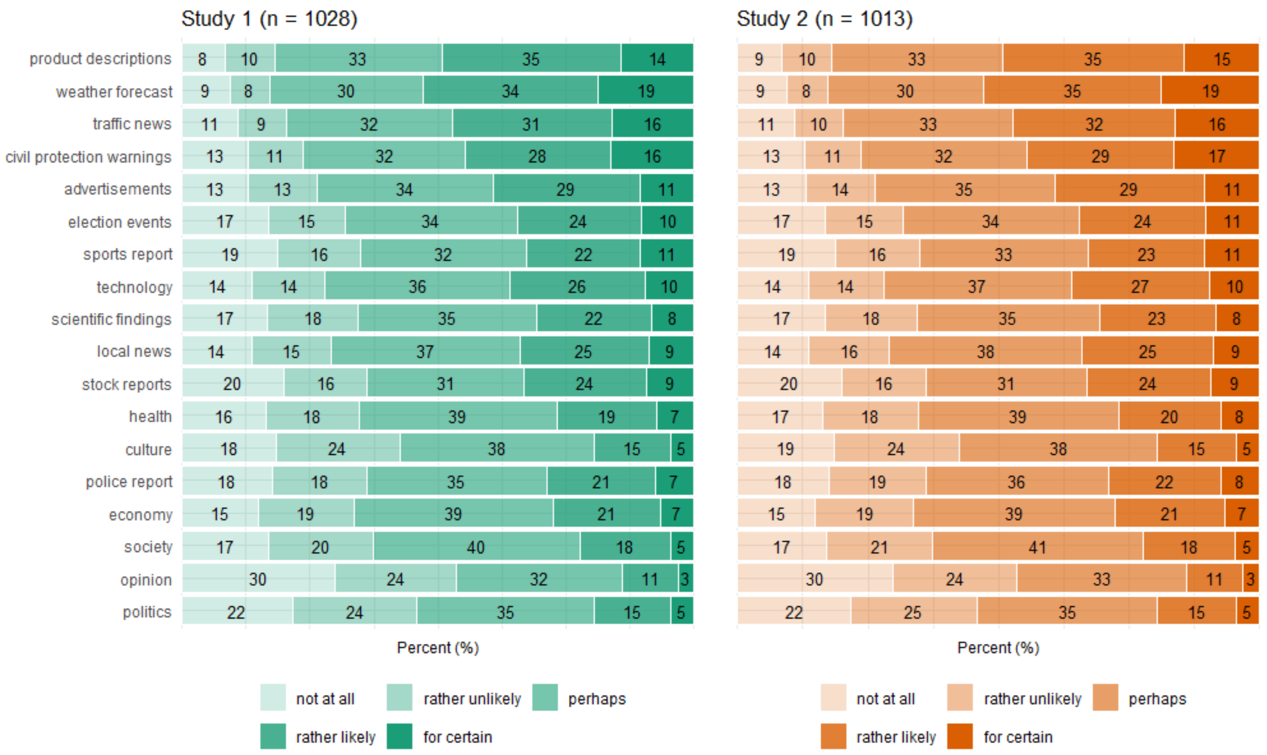

Supplementary Figure S1. Relative frequencies (percentage) of participants' perceived probability on how likely they would be to read 18 topics written by AI, by study.

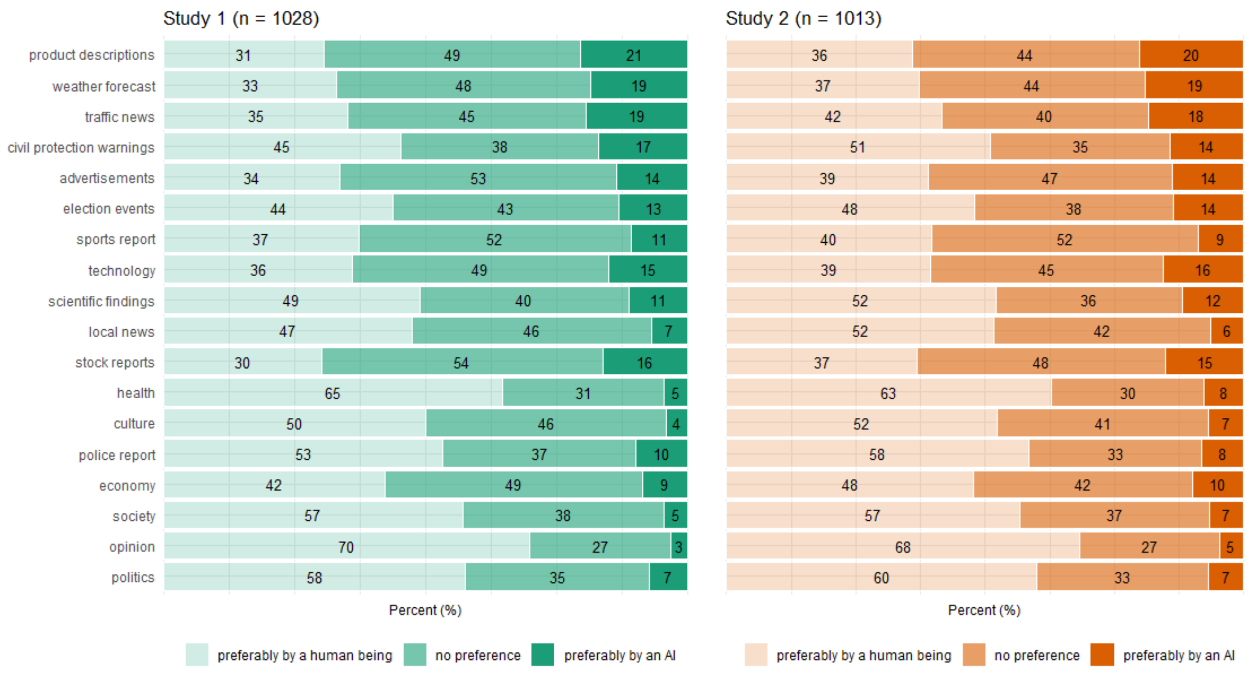

Supplementary Figure S2. Relative frequencies (percentage) of participants' author preference regarding 18 topics, by study.

Variable	Study 1 M (SD) 18-29 (*n* = 184)	30-39 (*n* = 182)	40-49 (*n* = 175)	50-59 (*n* = 232)	>60 (*n* = 255)	Study 2 M (SD) 18-29 (*n* = 188)	30-39 (*n* = 188)	40-49 (*n* = 188)	50-59 (*n* = 248)	>60 (*n* = 201)
Self-assessed knowledge	2.49 (0.86)	2.62 (1.06)	2.62 (0.97)	2.45 (0.87)	2.52 (0.93)	3.03 (1.03)	2.85 (1.01)	2.61 (0.96)	2.46 (0.98)	2.34 (0.86)
General attitudes	3.22 (0.53)	3.26 (0.62)	3.07 (0.62)	3.07 (0.66)	3.18 (0.71)	3.18 (0.59)	3.12 (0.66)	2.99 (0.69)	2.89 (0.70)	2.86 (0.74)
Machine heuristic	3.45 (0.65)	3.50 (0.68)	3.38 (0.80)	3.36 (0.79)	3.58 (0.71)	3.42 (0.70)	3.33 (0.80)	3.30 (0.76)	3.21 (0.85)	3.24 (0.80)
Knowledge	-	-	-	-	-	6.80 (3.09)	5.92 (3.30)	5.78 (3.13)	5.52 (3.69)	5.53 (3.37)
Performance expectancy	2.93 (0.72)	3.02 (0.79)	2.75 (0.76)	2.83 (0.89)	2.76 (0.89)	3.22 (0.85)	3.02 (0.85)	2.99 (0.83)	2.82 (0.90)	2.78 (0.98)
Effort expectancy	3.25 (0.67)	3.24 (0.75)	3.15 (0.73)	3.21 (0.73)	3.22 (0.80)	3.47 (0.76)	3.30 (0.79)	3.31 (0.71)	3.30 (0.80)	3.17 (0.85)
Attitude twd using ATG	3.11 (0.66)	3.11 (0.79)	2.87 (0.81)	2.95 (0.81)	3.01 (0.86)	3.21 (0.82)	3.12 (0.81)	3.03 (0.82)	2.92 (0.89)	2.91 (0.99)
Anxiety	2.71 (0.75)	2.69 (0.83)	2.80 (0.79)	2.74 (0.87)	2.55 (0.86)	2.88 (0.88)	2.90 (0.80)	2.74 (0.77)	2.73 (0.84)	2.76 (0.97)
Behavioral intentions to consume ATG	2.88 (0.72)	2.92 (0.79)	2.71 (0.82)	2.75 (0.83)	2.65 (0.91)	3.02 (0.85)	2.92 (0.88)	2.79 (0.85)	2.67 (0.96)	2.57 (0.95)
Permission	2.72 (0.79)	2.74 (0.66)	2.65 (0.73)	2.64 (0.74)	2.63 (0.69)	2.87 (0.74)	2.86 (0.67)	2.80 (0.71)	2.62 (0.74)	2.65 (0.73)
Attitude twd ChatGPT	-	-	-	-	-	3.28 (0.51)	3.22 (0.63)	3.15 (0.59)	3.07 (0.64)	2.95 (0.74)
Lay attitude twd ChatGPT & ATG	-	-	-	-	-	3.08 (0.63)	2.97 (0.65)	2.91 (0.72)	2.81 (0.79)	2.67 (0.76)

Supplementary Table S1. Means and standard deviations of all variables separated by age group and study.
